# Assessment of quality of care given to diabetic patients at Jimma University Specialized Hospital diabetes follow-up clinic, Jimma, Ethiopia

**DOI:** 10.1186/1472-6823-11-19

**Published:** 2011-12-20

**Authors:** Esayas K Gudina, Solomon T Amade, Fessahaye A Tesfamichael, Rana Ram

**Affiliations:** 1 Department of Internal Medicine, Jimma University, Jimma, Ethiopia; 2 Department of Internal Medicine, Jimma University, Jimma, Ethiopia; 3 Department of Epidemiology and Biostatistics, Jimma University, Jimma, Ethiopia; 4 Emergency Medicine Resident, St Barnabas Hospital. Bronx, NY

## Abstract

**Background:**

Sub-Saharan Africa is currently enduring the heaviest global burden of diabetes and diabetes care in such resource poor countries is far below standards. This study aims to describe the gaps in the care of Ethiopian diabetic patients at Jimma University Specialized Hospital.

**Methods:**

329 diabetic patients were selected as participants in the study, aged 15 years or greater, who have been active in follow-up for their diabetes for more than 1 year at the hospital. They were interviewed for their demographic characters and relevant clinical profiles. Their charts were simultaneously reviewed for characters related to diabetes and related morbidities. Descriptive statistics was used for most variables and Chi-square test, where necessary, was used to test the association among various variables. P-value of < 0.05 was used as statistical significance.

**Results:**

Blood glucose determination was done for 98.5% of patients at each of the last three visits, but none ever had glycosylated haemoglobin results. The mean fasting blood sugar (FBS) level was 171.7 ± 63.6 mg/dl and 73.1% of patients had mean FBS levels above 130 mg/dl. Over 44% of patients have already been diagnosed to be hypertensive and 64.1% had mean systolic BP of > 130 and/or diastolic > 80 mmHg over the last three visits. Diabetes eye and neurologic evaluations were ever done for 42.9% and 9.4% of patients respectively. About 66% had urine test for albumin, but only 28.2% had renal function testing over the last 5 years. The rates for lipid test, electrocardiography, echocardiography, or ultrasound of the kidneys during the same time were < 5% for each. Diabetic neuropathy (25.0%) and retinopathy (23.1%) were the most common chronic complications documented among those evaluated for complications.

**Conclusions:**

The overall aspects of diabetes care at the hospital were far below any recommended standards. Hence, urgent action to improve care for patients with diabetes is mandatory. Future studies examining patterns and prevalence of chronic complications using appropriate parameters is strongly recommended to see the true burden of diabetes.

## Background

Diabetes mellitus (DM) is one of the chronic illnesses with multi-system complications; the prevalence of which is alarmingly increasing [1,2]. It is often accompanied by various chronic complications that may affect the productivity and quality of life inevitably [3]. Today, at least in the western world, DM is the leading cause of blindness; non-traumatic amputation; and chronic renal failure [4]. Diabetes reduces life expectancy by 5 to 10 years. Premature cardiovascular disease is the most common cause of mortality [5].

The duration of diabetes, degree of hyperglycaemia, hypertension, dyslipidaemia, and smoking are the strongest risk factors for chronic complications of diabetes [5]. The risk of vascular complications can thus be greatly reduced by tight management of these risk factors [5-8]. In fact, diabetes care is complex and requires continuing medical care and patient self-management education [6].

The situation is far worse in the developing world [9]. Indeed, coronary heart disease is already the leading cause of mortality in some developing countries [10]. In sub-Saharan Africa in particular, the condition is even worse due to late diagnosis and poor access to diabetes care [9-12].

Diabetes is not uncommon in Ethiopia but the incidence and prevalence of the disease is not well known in the community. Limited studies have shown a significant increase in its prevalence over the last four decades [13-17], poor access to diabetes care [18], and high rates of chronic complications [19-26]. In one of the recent studies, accesses for blood glucose monitoring and diabetes health education were found to be very low. About half of the patients didn't have urine analysis, renal function and lipid test done in the previous 1-2 years and none ever had glycosylated haemoglobin (HbA1c) determination. About 75% of the patients required admissions directly or indirectly due to uncontrolled diabetes [18]. The cost of inpatient diabetes management in the country is enormous being significantly higher than the cost of other inpatient managements [27].

However, diabetes in Ethiopia has never been given the attention it deserves. Glycaemic control and management of co-morbid conditions and diabetes complications are alarmingly sub-optimal and perhaps one of the worst in the world [18]. Furthermore, the overall disease burden in the country is unknown due to very limited studies in the country. The available studies lack generalizability due to small sample sizes most of which were limited to the capital, Addis Ababa. Besides, there is no national strategy for the prevention and control of diabetes.

The aim of this study was to describe the current situation for a group of Ethiopian diabetic patients regarding diabetes specific characteristics, assessment of hypertension management and the occurrence of diabetes related morbidities.

## Methods

### Study design

a cross-sectional study design was used.

### Settings

This study was conducted from August 29 to November 2, 2009 at Jimma University Specialized Hospital (JUSH). JUSH is a teaching and referral hospital located in Jimma town, Southwest Ethiopia. Diabetes clinic is one of the many chronic follow-up clinics of the hospital occurring twice weekly on Mondays and Tuesdays. The service is rendered by internists, medical residents, medical interns, and general nurses (with no special training).

Frequency of follow-up visits is at least 3 times a year and depends on proximity to hospital and need for close follow-up. Patients with evidence of complications get more frequent check-ups. On each visits, patients are usually given diabetes health education by members of Ethiopian Diabetes Society, who are non-professionals and have diabetes themselves. On the follow-up day, all patients are expected to come with results of FBS done within 48 hours. Their charts are kept at diabetes follow-up clinic.

Participants of this study were diabetic patients who have been active in follow-up for their diabetes for more than 1 year at JUSH. At the time of the study there were a total of 1716 diabetic patients (both adult and paediatric patients) on follow-up care at the hospital. Of these, 1353 were older than 15 years and were taken as a target population from which study participants were selected.

### Selection of study participants

a sample size of 329 patients was obtained using a minimum sample size calculation. All eligible patients who were willing to participate in the study were scrutinized until the planned sample size was obtained. They were invited to participate in the study in consecutive order and their records were reviewed after their consents were obtained. For those who had repeated clinic visits during the study period, data were collected during their first visits. For the 19 patients who declined to participate in the study, no further information was obtained from them or their records.

### Data collection

The data were collected by using a structured questionnaire which was specifically prepared for this study and was compared with those used in previous studies done in the country. Patients were interviewed for their demographic features, characters related to diabetes and hypertension management where applicable, hospital admission, current symptoms, and their attitude towards care given to them. Their charts were reviewed for types of diabetes; types of treatments for diabetes and hypertension; last three records of blood glucose, BP, and weight; and causes of admission. Documented findings of laboratory data, ECG, imaging studies, eye evaluations, neurologic examinations, and diabetes complications were also sought from their medical records. Patients were interviewed in a separate room with complete privacy. Both the interviews and record reviews were done by medical interns and residents.

### Data quality control

to ensure the quality of data, data collectors were selected based on their interests and were adequately oriented and data collection was supervised by principal investigators. Information provided by patients for doses of drugs, treatment modifications, and clinical evaluations were cross checked from their records for consistency. The collected data were also checked for completeness and internal consistency.

### Data processing, analysis and interpretation

the data were coded, cleaned, entered and analysed with the help of SPSS window program version 16. Descriptive statistics was used for most variables and Chi-square test, where necessary, was used to test the association among various variables. P-value of < 0.05 was used as statistical significance. Average blood sugar, blood pressure and weight refer to the average of the last three measures.

### Ethical considerations

Ethical approval was obtained from Jimma University Ethical review board. Informed and written consent was obtained from each study participant. People with life threatening illnesses were referred for emergency management.

## Results

### Background Characteristics

The male to female ratio was 1.46:1. The mean age of patients at the time of the study was 48.4 ± 15.1 years with range of 15 to 82. The duration since DM diagnosis ranged between 1 and 41 years with a mean of 7.4 ± 5.2 years. Family history of DM was reported by a fifth of patients, with the proportion being higher among type 2 patients (Table 1).

**Table 1:** Background characteristic of diabetic patients on follow-up at JUSH, November 2009

Characteristics	Frequency	%
Age (years)		
15 - 34	62	18.8
35 - 64	212	64.4
≥ 65	55	16.7
Gender		
Male	195	59.3
Female	134	40.7
Occupation		
Unemployed	115	35.0
Farmer	93	28.3
Employed (paid work)	106	32.2
Student	15	4.6
Literacy status		
Illiterate	112	34.0
Non-formal education	14	4.3
Elementary	105	31.9
High school	57	17.3
Above high school	41	12.5
Type of diabetes		
Type 1	117	35.6
Type 2	212	64.4
Family history of diabetes		
Yes	67	20.4
No	262	79.6
Duration since diagnosis (years)		
1-5	157	47.7
6-10	106	32.2
≥ 11	66	20.1
Blood glucose on each visits		
Not done	5	1.5
FBS	312	94.8
RBS	12	3.6
Access for SMBG		
Yes	18	5.5
No	311	94.5
Access for drugs*		
Free	321	97.9
Paid	7	2.1

RBS - random blood sugar, SMBG - self monitoring of blood glucose, FBS - fasting blood sugar

*One patient was not taking any glucose lowering agent and was not included.

### Adequacy of glycaemic control

Blood glucose was measured for 98.5% of the patients during all of their last 3 visits. None of the patients ever had glycosylated haemoglobin test (Table 1). The average FBS was comparable among both types of diabetes with mean of 171.7 ± 63.6 mg/dl. Over 2/3^rd ^of both groups had a mean FBS above the target level of 130 mg/dl (Table 2).

**Table 2:** Patterns of glycaemic management among diabetic patients at JUSH, November 2009

Glycemic managements	Type 1 DM		Type 2 DM		Total	
	
	N	%	N	%	N	%
Initial management						
MNT	4	3.4	14	6.6	18	5.5
OGLA	15	12.8	188	88.7	203	61.7
Insulin	98	83.8	9	4.2	107	32.5
Herbal treatment	0	0.0	1	0.5	1	0.3
Current management						
MNT alone	0	0	1	0.5	1	0.3
Insulin alone	109	93.2	61	28.8	170	51.7
Insulin and OGLA	7	6.0	6	2.8	13	4.0
Single OGLA	0	0.0	69	32.5	69	21.0
Combination OGLA	1	0.8	75	35.4	76	23.1
Initiation of pharmacologic treatment						
At the time of diagnosis	112	95.7	184	86.8	296	90.0
Later on	5	4.3	28	13.2	33	10.0
FBS (mg/dl)						
< 70	0	0.0	2	1.0	2	0.6
70-130	24	22.4	58	28.3	82	26.3
> 130	83	77.6	145	70.7	228	73.1
Doses of drugs (*mean ****± ****SD*)						
Insuline - IU	52.4 ± 20.1		45.0 ± 15.8		49.7 ± 19.0	
Glibenclamide - mg/dl	20.0 ± 0		15.2 ± 6.5		15.3 ± 6.5	
Metformine- mg/dl	625.0 ± 231.5		882.0 ± 406.1		860.8 ± 400.1	

MNT - medical nutritional therapy, OGLA -oral glucose lowering agent, FBS - fasting blood sugar

Except for 1 patient, all were on pharmacologic therapy for their diabetes at the time of study. The delay in pharmacologic treatment after diagnosis was about 3 months and was longer in type 2 patients, 1 and 4 months for type 1 and 2 respectively(p < 0.01). At the time of the study, more than half of the patients were taking insulin alone or in combination with OGLA. The mean daily dose of glibenclamide was high with 54.0% of the patients taking at least 20 mg daily (Table 2).

Patients taking a single OGLA alone had a better glucose levels than those taking insulin alone or combination of OGLAs or insulin and OGLA (p = 0.0006). Patients taking lower doses of oral agents also had better blood sugar control than those taking higher doses. However, a similar trend did not occur for insulin. Among patients taking combination OGLA, 56.6% were taking glibenclamide ≥20 mg/d and metformin ≥1000 mg/d and over 90% of them had sub-optimal glycaemic control. Despite having FBS well above target level over the last three visits, no modification was done for glycaemic management for 69.3% of these patients (Table 3).

**Table 3:** Adequacy of glycaemic management in diabetic patients on follow-up at JUSH, November 2009

	FBS					
	
Treatment categories	Average FBS, mg/dl	≤ 130 mg/dl		> 130 mg/dl		P-value
			
		N	%	N	%	
Pharmacotherapeutic options						
Insulin alone	170.8	37	23.6	120	76.4	
Insulin and OGLA	190.3	3	23.1	10	76.9	0.0006
One OGLA	152.7	28	41.8	39	58.2	
Both OGLA	188.7	15	22.4	59	77.6	
Doses of drugs						
Insulin						
< 1IU/Kg/d	167.7	36	25.5	105	74.5	0.175
**≥ **1IU/kg/d	194.5	4	13.8	25	86.2	
Glibenclamide (G)						
< 20 mg/d	149.4	30	50.0	30	50.0	0.0001
**≥ **20 mg/d	190.0	11	15.1	62	84.9	
Metformin(M)						
< 1000 mg/d	163.1	15	38.5	24	61.5	0.001
**≥ **1000 mg/d	204.3	5	8.9	52	91.1	
Glibenclamide + metformin						
G < 20 mg/d or M < 1000 mg/d	168.1	12	36.4	21	63.6	0.005
G **≥ **20 mg/d & M **≥ **1000 mg/d	203.3	4	9.5	38	90.5	
Modification of glycaemic treatment during the last 3 visits						
Yes		16^*^	19.0	70	30.7	
No		68	81.0	158	69.3	

^* ^*In 13 of these patients treatment modification was for low FBS or symptoms of hypoglycemia.*

Level of glycaemic control was not significantly affected by sociodemographic characteristics of the patient, duration of diabetes, health education, and frequency of visits.

### Assessment of Adequacy of Hypertension management

The mean BP over the last three visits was 124.1 ± 17.2 and 80.7 ± 9.4 mmHg for SBP and DBP respectively. At the time of the study, 44.4% of patients had already been diagnosed to have hypertension. The majority (89.0%) of them were on pharmacologic treatments. The commonly used antihypertensive drugs were ACE inhibitors. About 75% of them were taking a single antihypertensive agent (Table 4).

**Table 4:** Patterns of Hypertension management in diabetic patients on follow-up at JUSH, November 2009

Characteristics	Frequency	(%)
History of hypertension		
Yes	146	44.4
No	183	55.6
Pharmacologic treatment for hypertension		
Yes	130	89.0
No	16	11.0
Proportion with hypertension by diabetes type		
Type 1 (*N = 117*)	30	20.5
Type 2 (*N = 212*)	116	79.5
BP in mmHg		
< 130/80	118	35.9
130/80 to < 140/90	110	33.4
140/90 to < 160/100	81	24.6
≥ 160/100	20	6.1
Antihypertensive agents		
HCT	16	12.3
ACE inhibitor	110	84.6
Beta blocker	10	7.7
Calcium channel blocker	21	16.2
Others	11	8.4
Option of drugs		
Single agent	95	73.1
Two drugs	32	24.6
Three drugs	3	2.3

BP - blood pressure, HCT - hydrochlorothiazide, ACE - angiotensin converting enzyme

Only 6.8% of patients already diagnosed to have hypertension had target BP level of <130/80 mmHg. Among patients who have never been diagnosed to have hypertension, 41.0% (75/183) had measurements higher than the target. Overall, 64.1% of the patients had BP higher than the target level at least over the last three visits. Despite having higher BP over the last three visits, only 21.8% of these patients had modification of their antihypertensive treatment (Table 5).

**Table 5:** Adequacy of Hypertension management in diabetic patients on follow-up at JUSH, November 2009

	BP (in mmHg)				
		
Characteristics	< 130/80		≥ 130/80		P value
		
	N	%	N	%	
History of hypertension					
Yes	10	6.8	136	93.2	0.0001
No	108	59.0	75	41.0	
Pharmacologic treatment of hypertension					
Yes	9	6.9	121	93.1	0.92
No	109	54.8	90	45.2	
Treatment modification of the last 3 visits					
Yes	3	2.5	46	21.8	0.599
No	115	97.5	165	78.2	

### Assessment of efforts done to watch for and prevent diabetes related morbidities

Only 68.1% of the patients had their weight measured on each visits of the last 3 with mean of 64.4 ± 12.1 Kg (59.3 ± 10.8 kg in type 1 and 67.1 ± 11.9 kg in type 2). No patient had his BMI ever done as there is no routine height measurement at the clinic (Table 6).

**Table 6:** Clinical follow-up patterns of diabetic patients at JUSH, November 2009

Characteristics	Frequency	(%)
**Physical examination**		
Weight measurements during the last 3 visits	224	68.1
Evaluation for DM neuropathy	31	9.4
Diabetic eye evaluation ever	141	42.9
Recent finding of eye evaluation		
Normal	36	25.5
Cataract/glaucoma	14	9.9
Diabetic retinopathy	26	18.4
No documentation	65	46.1
**Investigation over the last 5 years**		
RFT	97	29.5
Urinalysis	216	65.6
Lipid profile	16	4.9
ECG	13	4.0
Echocardiography	9	2.7
Ultrasound of the kidneys	13	4.0
**Treatments to prevent complications**		
Aspirin	46	14.0
Lipid lowering drugs	9	2.7
**DM related admissions during the last year**	**39**	**10.9**
DKA	28	71.8
Hypoglycemia	2	5.1
Ulcer or gangrene of extremities	5	12.8
Stroke	2	5.1
Other	2	5.1
**Documented complications**	**108**	**32.8**
DKA	44	40.7
Peripheral neuropathy	27	25.0
Diabetic retinopathy	25	23.1
Diabetic foot ulcer	11	10.2
Stroke/IHD	12	11.1
Others	7	6.5
**Current symptoms**	***247***	***75.1***
Blurring of vision	106	32.2
Numbness	100	30.4
Polysymptoms	74	22.5
Easy fatigability	71	21.6
Breathlessness/Chest pain/Body swelling	51	15.5
Foot ulcer/delayed wound healing	22	6.7
Impotence	15	4.6
Others	21	6.4

RFT - renal function test, ECG - electrocardiography, DKA - diabetic ketoacidosis, IHD - ischemic heart disease

Diabetes eye evaluations (visual acuity and ophthalmoscopic examination) were done for only 42.9%. Of them only 35.5% were sent as part of routine evaluation. Diabetic retinopathy was documented in 18.4% of them. Among 31 for whom neurologic examination was done, 28 (90.3%) had evidence of sensory polyneuropathy (Tables 6).

Only 29.5% (97) of the patients had renal function test (RFT) done over the last 5 years, 19.8% (19) of them had impaired renal function. Urinalysis was done for 65.7% of patients. The rates of proteinuria, glycosuria, and ketonuria were 28.7, 63.4, and 9.3% respectively (Table 6).

Over 10% of patients reported hospital admission over the past year due to diabetes related conditions. Diabetes related complications were documented in 32.8% of the patients. About 75% of them were complaining of DM related symptoms at the time of study (Table 6).

### Patients' satisfaction with diabetes care

Despite all the above findings, 95.3% (313) of the patients reported that they are satisfied with the care they are given at diabetic clinic of JUSH.

## Discussions

This study assessed a wide scope of diabetes care at Jimma University Specialized Hospital in Ethiopia using information from chart reviews and patients. Glycaemic control and blood pressure control were far below any recommended standards and attempts to prevent, detect early and manage chronic complications of diabetes were alarmingly poor. The mean FBS of 171.1 ± 63.6 mg/dl is better than the 190 ± 89.6 mg/dl in Addis Ababa [18] however; it is far higher than the recommendations in the developed world [6,28]. The majority of patients (73.1%) had FBS above the target level of 130 mg/dl as compared with 79% having >120 mg/dl in previous study [18] indicating that glycaemic control in Ethiopia is in dire need of being addressed. Similar to most studies in the country [18], no patient had HbA1c determination in this study because it is not available in public health sector in the country.

Over 99% of the patients were on pharmacologic treatment for their diabetes at the time of study with the delay after diagnosis for drug treatment being 3 months. Reasons may be lack of routine medical check-up and lack of knowledge of diabetic symptoms which resulted in patients presenting only when they were overtly sick.

Over 55% of the patients needed insulin and about 33% of type 2 patients have become insulin requiring during the course of their diabetes. This can be explained by the secondary insulin failure as the disease progresses since most patients were over 5 years post diagnosis. A similar trend has been documented in UK Prospective Diabetes Study (UKPDS) 33 in which case a significant proportion of patients on sulfonylurea subsequently required insulin due to severe hyperglycemia [7]. The daily insulin requirement was significantly higher in type 1 patients (52.4 Vs 45.0IU/Kg/d, p = 0.0001) which can be explained by the pathophysiology of the disease.

Patients taking a single oral agent were found to have a better glycaemic control than those taking insulin or combination OGLAs (p = 0.002). Possible explanation for this is the duration since diagnosis of diabetes as 72.5% patients taking single OGLA had diabetes for less than 5 years. In contrast, over 60% of patients requiring insulin and 56.6% of patients taking combination OGLAs had DM for over 5 years. This implies that good control in the single OGLA was due to the early disease course than the effect of the treatment given. A good illustration for this hypothesis is UKPDS 33 finding which showed progressive increments in fasting plasma glucose levels and doses of insulin and OGLA. During the follow-up period in that study, the median insulin dose increased by about 64% from 22IU at 3 years to 36IU at 12 years [7].

Similarly, patients taking lower doses of oral agents had a far better FBS level than those taking higher doses. More than 90% of patients taking glibenclamide > 20 mg/d and metformin > 1000 mg/d had persistently high FBS over the last 3+ visits. The mean FBS for them was 203.3 mg/dl and 65% of them had diabetes diagnosis for over 5 years. Surprisingly, over 80% of them did not have any modification in their glycaemic management over the last three visits. It is probably important here that patients taking such a high dose of oral agents are no more responsive to them and may sooner need insulin therapy [**7**]. Overall, a high FBS over the last 3 visits did not attract attention of treating physicians. In about 70% of the patients with high blood sugar, no modification in treatment regimen was done. Possible reasons are lack of awareness, time constraint, lack of adequate human power, and most importantly lack of appropriate guidelines and diabetes education for both care givers and patients.

Access for SMBG remains to be very low as it has been in previous study [18] (5.5 vs. 5.0%). However, access for blood glucose determination at the hospital was not found as a constraint as 98.5% of the patients had it done during each of the last three visits. Blood glucose determination is free at the hospital, but patients need to buy the glucometer and the strips for SMBG.

Morbidities and mortalities in patients having coexistence of hypertension and DM are immense [29,30]. Due to this fact, the target BP level for diabetes is consistently dropping and has become lower than the target level of the general population [6]. In previous studies in Ethiopia, hypertension was found to be an associated morbidity in 19.9% of diabetic patients as in Lester FT 1988 [31] and 34% Feleke Y 2005 [18]. However, the proportion of patients with hypertension in this study is much higher than the national findings and figures in the western world [29,30]. In this study, 44% patients have already been diagnosed with hypertension at the time of the study. Overall, 64.1% of the patients had systolic BP ≥130 mmHg and/or diastolic≥80 mmHg, and 24.6% had >140 and/or ≥ 90 mmHg.

About 90% of patients diagnosed to have hypertension were on pharmacologic therapy, the majority of which were taking ACE inhibitors, which abides with the general recommendation [6,32]. However, only 6.9% of patients currently taking antihypertensive medications had target BP. This figure, which does not look better than placebo effect, might have been due to under dosage, poor adherence to medications and lifestyle management, and less concern by health care providers.

The same as in the glycemic management, even though 100% of the patients had BP measurement on each visits; it was rarely used for patient management. Among patients not considered to have hypertension, 41.0% had mean BP of hypertensive ranges over the last 3 visits. Despite having BP of hypertensive range over the last three visits, 79.2% of the patients did not have modification to their hypertension regimen.

The proportion of patients who have been evaluated for diabetes related morbidities is very low. Less than 30% had RFT done and about 35% of patients did not have urinalysis within the last 5 years. Less than 5% of the patients had ECG, echocardiography, lipid test, or ultrasound of the kidneys. Alarmingly a significant proportion of tested patients had abnormal findings. Most of the imaging studies would have been expensive and inaccessible in the hospital setting; however, urine dipstick for albumin test is cheap and readily available at the hospital.

Far from recommended practice less than 10% of patients had ever had an evaluation for diabetic neuropathy. Those who had been examined were those with disabling symptoms as 90.3% of those evaluated had evidences for peripheral sensory polyneuropathy. Similarly, only 42.9% of patients in this study ever had a recommended yearly eye evaluation and the majority were evaluated for their symptoms rather than as a routine screening follow-up.

Diabetic ketoacidosis was found to be the commonest cause of hospital admission. Peripheral neuropathy and retinopathy were the most typical chronic complications identified. These findings are compatible with previous studies in Ethiopia [19] and other countries in Africa [22-26]. Similarly, the chief complaints for most patients were eye and sensory related. A significant proportion of them also had polysymptoms of diabetes that indicates poor sugar control.

Lifestyle management is an important component of diabetic care [6] and intensive nutrition treatment besides the conventional pharmacotherapy has been shown to improve both glycaemic control and anthropometric measures [33]. In this regard, diabetes nurse educators and diabetes dietitian play an important role in diabetic care. However, no emphasis has been given to diabetes health education at the clinic and in Ethiopia in general. To date there are no diabetes nurse educators and diabetes dietitian in the country. Those rendering health service for diabetes patients at the hospital had no special training for diabetes care and most of them were medical interns who were naïve not only to diabetes care but also to the general medical practice.

Demographic backgrounds, type and duration of DM since diagnosis, and duration of pharmacologic treatment of hyperglycemia were not found to affect level of glycaemic control. Similarly, the influences of good glycemic and hypertension control on morbidity were not observed in this study probably due to two major reasons. Firstly, most of the patients did not have adequate glycaemic and BP control. Secondly, documentation for diabetes related morbidities was found for only 1/3^rd ^of patients.

Most alarming is that there is very poor record keeping system and widespread trend of not documenting physical findings, laboratory results, and reason for changing managements. Despite this, the majority of the patients reported that they were satisfied with the care they were given at the clinic indicating that patients have inappropriate expectation about diabetes care. Poor awareness among patients about the extent and components of diabetes care affects not only their expectations but importantly the quality of services they obtain and the outcome of diabetes. In this study it may be mentioned here that patients might have been satisfied with the free laboratory services (for blood glucose) and medications, which were probably their main expectations of the care.

The strengths of our study was the high patient response rate, good cooperation from hospital staff, and the fact that it was one of the few studies on diabetes care in Ethiopia.

There are potential shortcomings in our study that require comment. The major limitation was poor chart keeping that might have limited us from getting full information about chronic complications of diabetes. Another limitation was the cross-sectional study design that is not adequate to assess most of the chronic complications of diabetes. The classification of diabetes to type 1 and type 2 was based solely on history and age of the patient. In this study, the proportion of type1 was 35.6% which is much higher than findings in the western world of 5 -10% [6]. We thus think that the proportion of type 1 diabetes may be lower than our finding with appropriate antibody study.

## Conclusions

Glycaemic and hypertension controls in diabetic patients at JUSH were far below any recommended standards and attempts to prevent, detect early, and manage chronic complications of diabetes were alarmingly poor. Besides these, inadequate knowledge and perception of patients about the scope of diabetic management and trends of poor documentation at the clinic were major constraints identified affecting over all care of diabetes patients.

Thus, we strongly recommend that urgent action should be taken to improve the management of such an alarmingly increasing morbidity that, if not acted upon, is debilitating to the patient and heavy burden to the economy of the country in particular. Due emphasis should be given to clinical examination and urine albumin test to screen for chronic complications of diabetes which are highly cost effective in such resource constraint settings. Available information should also be carefully interpreted and the findings be acted upon timely and appropriately. The documentation systems not only for diabetic patients but also for the general medical practice should improve so that patients will obtain an optimum and timely care. Further clinical investigations for adequacy of diabetes care and diabetes complications with appropriate parameters are also warmly recommended.

## Competing interests

The authors declare that they have no competing interests.

## Authors' contributions

EKG designed the study, developed instruments, supervised data collection and data entry, analyzed data and wrote manuscript. ST and FA participated in the study design, supervised instrument development, reviewed analysis and contributed to manuscript editing. RR participated in the revisions of the manuscript. All authors have read and approved the final manuscript.

## Pre-publication history

The pre-publication history for this paper can be accessed here:

http://www.biomedcentral.com/1472-6823/11/19/prepub
